# Parent supervision attributes profile questionnaire (PSAPQ) for young children: psychometric properties of the Chinese version

**DOI:** 10.1186/s12889-019-7362-4

**Published:** 2019-09-02

**Authors:** Jun Yang, Shih-Yu Lee, Yuqiu Zhou, Yuxia Cui, Yongkui Han, Hongling Song, Hui Zhang

**Affiliations:** 1 School of Nursing, Harbin Medical University (Daqing), No. 39 Xinyang Road, Gaoxin District, Daqing, 163319 Hei Longjiang Province China; 2 University of Texas at Tyler, 3900 University Blvd, Tyler, TX 75799 USA; 3 English Department, Harbin Medical University (Daqing), Daqing, Hei Longjiang Province China

**Keywords:** Parent supervision, Parent supervision attributes profile questionnaire, Reliability, Validity, Children

## Abstract

**Background:**

In China, thousands of children die from unintentional injury each year: the incidence rate of injury is from 19.4 to 64.3% which is the leading cause of mortality for children. An important factor to injury may be inadequate supervision. Thus, a linguistic and culturally appropriated, validated instrument to measure the supervision of children in Chinese primary caregiver is important and necessary. The purpose of this study was to translate and test the psychometric properties of the Chinese version of the Parent Supervision Attributes Profile Questionnaire (C-PSAPQ).

**Methods:**

This is a two-phase study. In phase I, the C-PSAPQ was produced by for- and back-ward translation. A total of 296 primary caregivers of 3–6 years old children were invited to participate in the second phase of the psychometric study. In order to assess the reliability of the C-PSAPQ, internal consistency and test-retest methods were performed. Additionally, construct validity was examined by using confirmatory factor analysis (CFA). The averaged variance extracted (AVE) and Bootstrap were used to test the convergent and to discriminate validity. The concurrent validity was assessed by evaluating the association between the self-reported C-PSAPQ and naturalistic observations.

**Results:**

The Cronbach’s α and intraclass correlation coefficients were acceptable for the C-PSAPQ and four subscales. The CFA supported a 4-factor loading model; however, the convergent validity was not acceptable (AVE < .5 for two subscales). The concurrent validity was supported.

**Conclusions:**

Due to the unacceptable convergent validity of the C-PSAPQ, an exploratory factor analysis is needed to ensure that the same trait is measured by its indicators in different cultures.

## Background

In China, thousands of children die from unintentional injury each year; the incidence rate of injury is from 19.4 to 64.3% and is the leading cause of mortality for children between the ages one and 14 [1]. Unintentional injury is a major cause of hospitalization and death for children in China and elsewhere; moreover, it is a heavy burden for society [2–4]. Most of the injuries are preventable since studies show that inadequate supervision is an important contributing factor to child injury [5–10]. Appropriate supervision could prevent children from attempting unsafe activities and assist them to accomplish tasks successfully [11]. When parents overestimated their children’s coping skills for unsafe environments that often put the children in danger; on the contrary, if parents underestimated the coping skills, they could become more protective and obstruct their children’s development of essential skills for safety and autonomy [12]. It is difficult to obtain the data regarding parental supervision; to date, methods including naturalistic observation, self-report about supervision, and event participation monitoring methods were used, all of which are labor and time consuming [5–9, 13–18].

Morrongiello [15, 16] developed the Parent Supervision Attributes Profile Questionnaire (PSAPQ) to assess parental protectiveness and supervision of children from parental interaction with the observational measures. The PSAPQ has 29 items, including four subscales: protectiveness, supervision beliefs, tolerance for children’s risk taking, and belief in fate. The psychometric properties of PSAPQ were tested by using 192 parents who had children aged 2–5 years old [15]. The results showed that the subscales were representative of four constructs by using confirmatory factor analysis, and the internal consistency (Cronbach’s α) was good for the subscales: protectiveness (*α* = .78), supervision beliefs (*α* = .77), tolerance for children’s risk taking(*α* = .79), and belief in fate (*α* = .78). In addition, the test-retest reliability for the four subscales were all acceptable (r = .72–.80, *p* < .001). Intercorrelations was significant between parents’ self-reported PSAPQ and the naturalistic observation by researchers. Further, convergent and discriminant validity were tested, and all indicated good construct validity. Correlations among the four subscales were within the acceptable range, for example, protectiveness and tolerance for children’s risk taking (r = − .37), protectiveness and belief in fate (r = − .13), and supervision beliefs and belief in fate (r = − .21); the results showed the factors were significantly distinct in different constructs. The PSAPQ has been widely used to assess supervision and children’s risks of unintentional injury in different studies with other children’s age groups, and all showed good psychometric properties [13–17].

In China, there is a specific type of family structure not found elsewhere in the world, which is called left-behind children. In this type of family structure, the parents are away from their hometown for work while their children stay in the hometown with grandparents. Prior studies revealed that single-parent children and left-behind children’s families are more vulnerable than the two-parent families for unintentional injury because of a lack of supervision [19–21]. However, controversial findings pointed out that caregivers who spent more time taking care of their children couldn’t decrease the unintentional injury; instead, the quality of supervision matters [20, 22]. Therefore, a properly validated instrument to measure the supervision of children among Chinese primary caregivers is important and necessary. Currently, there is no available measurement of parental supervision in Chinese language. Thus, the aims of this study were to translate and adapt the PSAPQ into Chinese (C-PSAPQ), and further to test the reliability, validity, and factor structure of the C-PSAPQ in Chinese primary caregivers of 3–6 years old children.

## Methods

### Study design

The ethics of this study was approved by Harbin Medical University. Two phases were conducted in the study. First, the original questionnaire was adapted and translated into Chinese, and, second, was the psychometric testing phase.

#### Phase 1: translation and adaption of the PSAPQ

In adapting the PSAPQ to this study, translation theory and the recommended procedures for cross-cultural research were used to forward-translate the instrument into Chinese and then the Chinese version was back-translated into English using the criteria developed by Flaherty and colleagues to assure semantic, contextual, and technical equivalence of the original and translated versions [23–27]. The panel experts included one Chinese American professor with bicultural backgrounds, two Chinese pediatric nursing teachers who were familiar with both English and Chinese, two college English teachers, and one fifth-grade Chinese language teacher.

A pretest was done with 10 stratified randomly selected primary caregivers of young children (3 to 6 years old), no further modification was needed. The errors in meaning were examined at the final review between the back-translated English version and the final Chinese version [24, 25].

#### Phase 2: testing phase

##### Participants and data collection

The study participants were recruited from four kindergartens (children aged 3 to 6) in the city of Daqing, Hei Longjiang Province in China. Data were collected from the school activities meeting when the parents were together with their children in the kindergarten from May 2017 to October 2017. Only one primary caregiver from each family was allowed in this study, and the inclusion criteria for the primary caregiver were: (1) live with the participating child at least 50% of the time and be the main caretaker of the child, (2) able to read and write in Chinese. Exclusion criteria included children with congenital medical problems and children with behavioral problems (e.g., autism, ADHD, schizophrenia), which was screened by using the Child Behavior Rating Scale (CBRS) [28]. Any children who scored 10 and above on the CBRS were excluded, and a referral was made for further evaluation.

The written informed consent was obtained from each study participant. The participants were instructed to fill out the questionnaire at home and return it to the kindergarten teacher. A total of 322 primary caregivers participated in the study, and 296 of them completed the study (response rate was 92%). Among them, 50 primary caregivers were invited back, three weeks after, for the test-retest reliability study.

### Measures

#### The family information and unintentional injury history form

The author (HZ) developed this form to collect the primary caregiver’s sociodemographic information and the child’s past year unintentional injury history, which derived from the International Classification of Disease (ICD-10) [29]. Unintentional injury was defined as an injury that (a) was diagnosed as a non-fatal injury by physicians and received medical treatment or (b) received emergency medical treatment or assistance from teachers, parents or others, and (c) required the child to rest for more than half a day before returning to normal activity [30]. The primary caregivers were asked to list the frequency of injury and to rate the severity of each injury.

#### The Chinese version of the parent supervision attributes profile questionnaire (C-PSAPQ)

The C-PSAPQ contains 29 items covering four dimensions: protectiveness with 9 items, supervision beliefs with 9 items, tolerance for children’s risk-taking with 8 items, and belief in fate with 3 items. Caregivers were asked to rate each item using a five-point Likert-type scale (1 = never to 5 = all of the time). A higher total scores indicated the primary caregivers had more engagement with supervision for their children.

#### The naturalistic observations

Morrongiello and House [16] used naturalistic observation to examine the relevance between parental attributes that measured from the PSAPQ and behaviors, a similar protocol of naturalistic observations was adopted in the current study. A Likert-type checklist with five dimensions was used in the naturalistic observations. The five dimensions are: visual supervision (3 = watching child continuously, 1 = not at all), auditory supervision (3 = able to hear the child continuously, 1 = not at all), physical proximity (5 = constant physical contact with child, 1 = beyond reach of the child), parent distraction (5 = parent is completely focused on child, 1 = all the parent’s time is spent on distraction activities), and parent engagement with child (4 = all the parent’s time was spent actively playing with child, 1 = completely uninvolved and inattentive to the child’s play) [16]. Collapsing scores provided a total supervision score for each behavior. Higher scores indicate parents pay more attention to the children in the specific setting.

In the pilot study, ten parent-child dyads were individually involved in a 10-min naturalistic observation on the kindergarten playground, and the parents subsequently completed the self-report C-PSAPQ, and the naturalistic observation checklist [15]. While the parent-child dyads in the playground, the first author (HZ) and a trained kindergarten teacher, were simultaneously using the observation checklist to evaluate parental supervision. The inter-rater reliability was established before they conducted the field observation. The two observers simultaneously stayed close by each of the parent-child dyad participants in the playground and naturally observed them. After the 10-min observation, the two observers compared their assessments with each other to reach an agreement, then collapsed the agreed scores for the supervision scores. The scores of the total naturalistic observation and its five dimensions from the researchers then compared with the parental self-reported scores, which showed highly correlated with each other (r = .75–.92, *p* < .001).

### Statistical analysis

Statistical analysis was performed by SPSS 18.0 and AMOS 20.0. Descriptive statistics were used for analyzing the demographic characteristics. Mean and standard deviation were used to analysis the interval and ratio data, such as age, severity of injury; frequency statistics were used to examine nominal data such as sex, marital status, and education. Psychometric properties of the C-PSAPQ were measured by the following statistical analyses.

### Reliability

The reliability of the C-PSAPQ was assessed by the internal consistency and test-retest reliability. Intraclass correlation coefficients (ICC) were applied to assess the test-retest reliability, and the split in half reliability was also tested. The values for Cronbach’s alpha coefficient and ICC equal to .60 or higher were considered acceptable [14, 31].

### Validity

Construct and concurrent validity were measured. Confirmatory factor analysis (CFA) was used to measure the construct validity and to determine if the factors in the original study were supported in the current Chinese population. For the CFA, model fit was evaluated by using multiple fit indices including the ratio Chi-square and degrees of freedom (*χ*^2^/*df*), Goodness of Fit Index (GFI), Adjusted Goodness of Fit Index (AGFI) and Root Mean Square Error of Approximation (RMSEA). The *χ*^2^/*df* < .3, GFI > .9, AGFI> .9, and RMSEA < .08 were accepted [32]. Based on the corrected correlations from the CFA model, the averaged variance extracted (AVE) and the Bootstrap ML were used to test the convergent and discriminate validity [33].

Concurrent validity was evaluated by the correlation between the supervision score from naturalistic observation and the scores from PSAPQ and its subscales. In our pilot study, the supervision scores were significantly correlated between researcher’s and parental naturalistic observation. A decision, based on feasibility, was made to only include parental self-report supervision scores in the concurrent validity test. In addition, a standardized written scenario of child’s activity in playground instead of a real field observation for parents was provided for the naturalistic observation assessment.

## Results

A total of 296 caregiver-child dyads (Table 1) participated in this study, with a mean age of 59.1 (SD = 5.4) for caregivers and 5.3 (SD = 1.4) for children (163 boys and 133 girls). The majority of the study participants came from nuclear families (62.8%), with an average annual income of 50,000 yuan (55.7%) which is above the national average in China. More than half of the caregivers were college educated (65.5%), and were grandparents (63.9%). The frequency of injury in the past year was 113 (38.2%) never had any injury, 38 (12.8%) had one, 40 (13.5%) had twice, and 105 (35.5%) had more than three times. The average of severity of injury was 1.3 (SD = 0.8, ranged from 0 to 10).

**Table 1:** Characteristics of children and the primary caregiver

Characteristic (*n* = 296)	Value
Child mean age (SD)	5.3 (1.7)
Child sex, *n (%)*	
Boy	163 (55.1)
Girl	133 (44.9)
Primary caregiver for child, *n (%)*	
Parent(s)	106 (35.8)
Grandparent(s)	189 (63.9)
Babysitter and others	1 (0.3)
Primary caregiver mean age (SD)	59.1 (5.4)
Marital status of primary caregiver, *n (%)*	
Married	292 (98.6)
Divorced/Single	4 (1.4)
Education, *n (%)*	
Illiteracy	1 (0.3)
Under Junior High School	44 (14.9)
Senior High School	57 (19.3)
Above College	194 (65.5)
Employment status, *n (%)*	
Employed	92 (31.1)
Unemployed	20 (6.8)
Retirement	184(62.1)
Family Type, *n (%)*	
Nuclear family	186 (62.8)
Remarried family	6 (2.0)
Single-parent family	3 (1.0)
Extended family	101 (34.1)
Household income (per person per month in yuan), *n (%)*	
< 1000	3 (1.0)
1000–2000	11 (3.7)
2000–3000	35 (11.8)
3000–4000	82 (27.7)
> 4000	165 (55.7)
Religious faith, *n (%)*	
Yes	9(3)
No	287(97)
Frequency of injury (per year)	
None	113(38.2)
Once	38(12.8)
Twice	40(13.5)
More than three times	105(35.5)
Severity of injury (SD)	1.3(0.8)

The total score for the C-PSAPQ was 101.36 (SD = 13.77); for the four subscales were: 34.30 + 5.02 (protectiveness), 33.71 + 5.56 (supervision beliefs), 28.42 + 5.61 (tolerance for children’s risk taking), and 4.96 + 2.25 (belief in fate). Compared to the findings from Petrass’ study [13], the family caregivers in the current study scored significantly higher in protectiveness (M = 19.3; t = 51.38, *p* < .01), supervision beliefs (M = 18.3; t = 47.67, p < .01), and tolerance for children’s risk taking (M = 22; t = 19.67, p < .01). However, the mean score of belief in fate was significantly lower than Petrass’ results (M = 13.1; t = − 62.16, p < .01).

### Reliability

The Cronbach’s α for the C-PSAPQ was .84 and ranged from .61 to .78 for its subscales. In the test-retest analysis, the ICCs from the 50 parents were .92 for the C-PSAPQ and ranged from .82 to .87 for the subscales (Table 2). The split in half reliability of C-PSAPQ was .80.

**Table 2:** Compared reliability of the Chinese and original version of PSAPQ

Subscale (items)	Cronbach’s α (*n* = 296)	ICC (*n* = 50)
Protectiveness		
Original version	0.78	
Chinese version	0.72	0.87
Supervision beliefs		
Original version	0.77	
Chinese version	0.64	0.82
Risk tolerance		
Original version	0.79	
Chinese version	0.78	0.85
Fate beliefs		
Original version	0.78	
Chinese version	0.61	0.86
Overall scale		
Original version		
Chinese version	0.84	0.92

### Validity

#### Construct validity

**Fig. 1:**
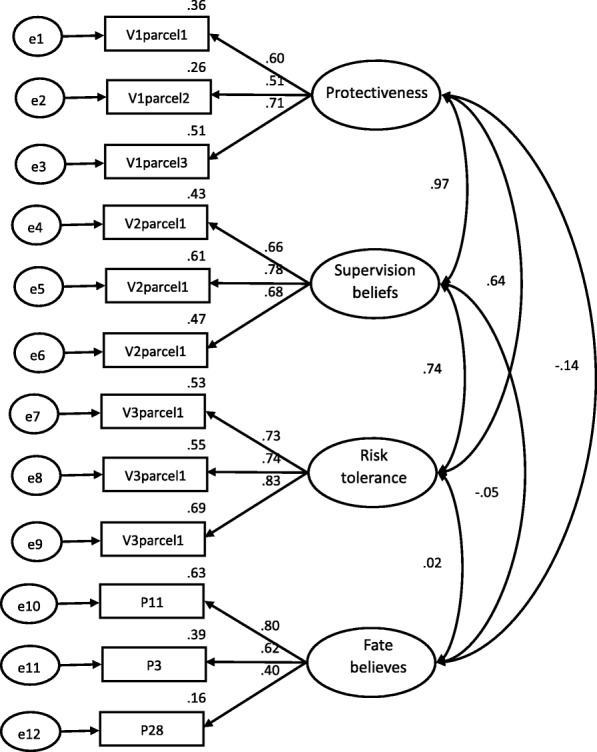
Results of the confirmatory factor analysis of C-PSAPQ

The CFA (*χ*^2^/*df* =1.14, CFI = .95, GFI = .95, RMSEA = .02, *P* = .002) confirmed.

the same four factors addressed by Morrongiello [34]. The diagram and values of the adjusted model were shown in Fig. 1 and Table 3.

**Table 3:** Parcel structure by item between original version and Chinese version of PSAPQ

		Factor Scores	
Factor	Parcel	Original V	Chinese V
Protectiveness			
Parcel 1		0.75	0.60
	I feel very protective of my child		
	I think of all the dangerous things that could happy		
	I keep my child from playing rough games or doing things where he/she might get hurt		
Parcel 2		0.71	0.51
	I make him/her keep away from anything that could be dangerous		
	I feel fearful that something might happen to my child		
	I warn him/her about things that could be dangerous		
Parcel 3		0.67	0.71
	I keep an eye on my child’s face to see how he/she is doing		
	I feel a strong sense of responsibility		
	I try things with my child before leaving him/her to do them on his/her own		
Supervision			
Parcel 1		0.83	0.66
	I have my child within arm’s reach at all times		
	I know exactly what my child is doing		
	I can trust my child to play by himself/herself without constant supervision		
Parcel 2		0.84	0.78
	I stay within reach of my child when he/she is playing on the equipment		
	I keep a close watch on my child		
	I say to myself that I can trust him/her to play safely		
Parcel 3		0.64	0.68
	I stay close enough to my child that I can get to him/her quickly		
	I hover next to my child		
	I make sure I know where my child is and what he/she is doing		
Risk tolerance			
Parcel 1		0.70	0.73
	I encourage my child to try new things		
	I let him/her learn from his/her own mishaps		
Parcel 2		0.87	0.74
	I let my child take some chances in what he/she does		
	I let my child do things for him/herself		
	I let my child experience minor mishaps if what he is doing is lots of fun		
Parcel 3		0.74	0.83
	I let my child make decisions for himself/herself		
	I encourage my child to take risks if it means having fun during play		
	I wait to see if he/she can do things on his/her own before I get involved		
Fate			
Item			
11	When my child gets injured it is due to bad luck	0.61	0.80
3	Whether or not my child gets injured is largely a matter of fate	0.71	0.62
28	Good fortune plays a big part in determining whether or not my child gets injured	0.92	0.40

#### Convergent-discriminate validity

Convergent validity was examined by average variance extracted (AVE) and composite reliability (CR). AVE measures the level of variance captured by a construct versus the level due to measurement error; a level of 0.5 and above is acceptable. CR could provide less biased estimates of reliability, and a value of 0.7 and above is acceptable [35]. The results of convergent validity (Table 4) revealed that two subscales, protectiveness and belief in fate, had unacceptable AVE and CR. The Bootstrap ML [36] was used to test discriminate validity, and the results were presented in Table 5. The confidence interval (CI) of paired correlation of two subscales (supervision beliefs and protectiveness) contains 1.0 (.88 to 1.06) which indicated that the two subscales could not discriminate with each other when the paired correlations of other subscales were below 1.0.

**Table 4:** The convergent validity of the PSAPQ

Regression		SE	t	Standardized	CR	AVE
V1parcel1←	F1			0.60	0.64	0.38
V1parcel2←	F1	0.11	7.25*	0.51		
V1parcel3←	F1	0.12	8.79*	0.72		
V2parcel1←	F2			0.66	0.75	0.50
V2parcel2←	F2	0.11	10.91*	0.78		
V2parcel3←	F2	0.11	9.52*	0.68		
V3parcel1←	F3			0.73	0.81	0.59
V3parcel2←	F3	0.08	11.43*	0.74		
V3parcel3←	F3	0.10	11.76*	0.83		
P11←	F4			0.80	0.65	0.40
P3←	F4	0.18	4.77*	0.62		
P28←	F4	0.13	4.45*	0.40		

Note: * means *p* < 0.001, V1 = Protectiveness, V2 = Supervision beliefs, V3 = Risk tolerance, V4 = Fate believes

**Table 5:** Correlation matrix of factors in PSAPQ

	Protectiveness	Supervision beliefs	Risk tolerance
Protectiveness	1		
Supervision beliefs	0.97** (0.88, 1.06)	1	
Risk toleranc	0.64** (0.51, 0.76)	0.74** (0.64, 0.82)	1
Fate believes	−0.14(−0.33, 0.05)	−0.05(−0.26, 0.11)	0.02(−0.19, 0.19)

Note: Show only diagonal and lower left half correlations matrix; 95% confidence interval in parentheses; ** *p* ≤ 0.01

#### Concurrent validity

To assess the concurrent validity of the C-PSAPQ, the correlations between the parental self-report naturalistic observations and the scores of the C-PSAPQ and its subscales were assessed. There were statistically significant associations between the naturalistic observation and C-PSAPQ whole scale (r = .170, *p* < .01) and its two subscales, protectiveness (r = .205, p < .01) and supervision beliefs (r = .246, p < .01). No significant association was found with the other two subscales, tolerance for children’s risk taking and belief in fate. The results were similar to those from pilot study that the total score of naturalistic observation had low correlation with total score of C-PSAPQ and its subscales (r_s_ = −.34–.49, *p* > .05), with an exception in supervision beliefs (r_s_ = .849, *p* < .001).

## Discussion

The purpose of this study was to adapt the C-PSAPQ and assess the psychometric properties of this scale among the Chinese population. Findings from this study show that the C-PSAPQ is linguistically relevant to Chinese family caregivers for young children. As we translated the questionnaire across culture, no item had great modification to the Chinese cultural content. The C-PSAPQ may covers protectiveness, supervision beliefs, tolerance for children’s risk taking, and belief in fate.

The C-PSAPQ had acceptable internal consistency reliability, all above .80, which is in accordance with previous studies of this scale [11, 14]. The Cronbach’s α for the two subscales, supervision beliefs (α = .64) and belief in fate (α = .61), was less than previous reports (Supervision = .77, Fate = .78), but was still acceptable [25]. This might be related to the differences between Eastern and Western parents in differing socioeconomic, cultural, and religious factors, which affect the perception of the ability to care for children and attendant dangers. Recently, a few published studies suggest cultural differences may contribute to race differences in injury of the children [26, 28, 37]. The western culture encourages child risk-taking, adventures and impulsion. Many caregivers in the Western families expressed self-confidence in supervision and a belief that the safety of the children is a matter of luck or fate [6, 14, 37]. Some parents also believe that childhood injuries are normal and that they do not have the ability to prevent their children from injury, so they might pay less attention than parents who had higher self-confidence in controlling for injuries [38, 39]. However, the caregivers in Latin American and Asian families do not encourage their children’s risk-taking behaviors in order to protect them from unintentional injury [40, 41]. In China, it is quite easy for Chinese parents to blame themselves if the child has an injury because most Chinese parents have no religious faith; they believe that if the injuries happen they result from the parent’s negligent care for the children [42]. In the current study, almost all primary caregivers (97%) had no religious faith; most of them think they are masters of themselves instead of victims of destiny. Various researches showed parental education related to pediatric injury: the degree of supervision varied across different educational levels. Those caregivers with high educational levels accurately supervised to assess the risk level and to cope with the risk [14, 41]. In the current study, there are 194 (65.5%) caregivers with at least college level education, and the mean score of belief in fate was 4.96 which was lower than Petrass’ results [13]. This indicated that most caregivers had the confidence to supervise and keep their children safe. On the contrary, the scores of protectiveness, supervision beliefs and tolerance for children’s risk taking in this study are higher than Petrass’ study [13]. The results show that caregivers were inclined to pay more attention to children in order to avoid injuries. Although the mean score and the range for the extended version of the PSAPQ have been reported [13, 15, 34], to date, normative scores for the PSAPQ have not been published. The norms and cut-off scores for the PSAPQ should be further explored to identify children who had higher risk of injury as a result of lack of parental supervision.

Multiple methods were used in this study to assess the validity of the C-PSAPQ. The results from the CFA indicate that this instrument includes four dimensions just as Morrongiello originally proposed [34]; however, the loading to each factor was different when compared to the original version (Table 3). Although CFA confirmed the C-PSAPQ included the same four factors as Petrass’ study [13], the two subscales, protectiveness and supervision beliefs, are highly correlated with each other, which was also established in a study in Portuguese [14]. Since the exploratory factor analysis (EFA) was not performed in this study, further study should include EFA since the constructs might be comprehended differently in different cultures. Regarding the concurrent validity, although the C-PSAPQ scores were significantly correlated with the naturalistic observations, the correlation was relatively low, which indicates concurrent validity should be further explored.

To our best knowledge, this is the first study to test the psychometric evaluation of the C-PSAPQ in Chinese. Although we found sound psychometric properties, some limitations need to be considered. First, the purpose of this study was to test the psychometric properties of the C-PSAPQ; however, the convergent/discriminant validity in the constructs of supervision beliefs and protectiveness were ambiguous in this study. Therefore, further validation is needed, particularly, the study participants were from four kindergartens in the city of Daqing; thus, the findings might not be presented to other areas of China. It is necessary to increase a large sample size in a future study and to employ psychometric test of the C-PSAPQ in both urban and rural areas. Second, the majority of the caregivers’ educational levels in the present study was above college (65.5%) and most of the primary caregivers are grandparents (63.9%). The different types of supervision between grandparents and parents should be further assessed since the parenting style could be different between generations. In urban China, the three-generation family is very common; children are cared for by both their parents and grandparents who all pay much attention to the children in order to avoid injuries [43]. In contrast, the left-behind children were solely cared for by the grandparents; therefore, the injury prevalence rate might be different across the types of family structures and warrant further study. Third, although the concurrent validity had a statistical significance, the correlation was low in this large sample size, which either indicated the self-report questionnaire could not replace the observation method to predict unintentional injuries or it is problematic when comparing the association of the assessments between parents and the researchers. Fourthly, the participants of this study were all physically and mentally healthy children, and behavioral problems were excluded; the study could not provide a full explanation regarding parental supervision of children who would need additional attention. Further study should examine the characteristics of parental supervision and behaviors in the group of children with ADHD.

## Conclusion

Parental supervision is important for preventing child injury, but, to date, there is no reliable questionnaire to measure parental supervision in China. This study has shown that the C-PSAPQ has acceptable reliability, construct validity, concurrent validity, but not convergent/discriminant validity. Due to the unacceptable convergent/discriminant validity (supervision beliefs and protectiveness) of the C-PSAPQ, an exploratory factor analysis is needed to ensure that the same trait is measured by its indicators in different cultures in a further study.

## Data Availability

The datasets used and/or analyzed during this study are available from the corresponding author on reasonable request.

## Abbreviations

AGFI: Adjusted Goodness of Fit Index
AVE: averaged variance extracted
CFA: Confirmatory factor analysis
CFI: Conparative fit index
CI: Confidence interval
CVI: Content validity index
GFI: Goodness of fit index
ICC: Intraclass correlation coefficient
PSAPQ: Parent Supervision Attributes Profile Questionnaire
RMSEA: Root mean square error of approximation
